# Genome-wide capture sequencing to detect hepatitis C virus at the end of antiviral therapy

**DOI:** 10.1186/s12879-020-05355-2

**Published:** 2020-08-26

**Authors:** Peng Peng, Yanjuan Xu, Michael W. Fried, Adrian M. Di Bisceglie, Xiaofeng Fan

**Affiliations:** 1 Division of Gastroenterology & Hepatology, Department of Internal Medicine, Saint Louis University School of Medicine, St. Louis, MO 63104 USA; 2 Wuhan Pulmonary Hospital, Wuhan, 430030 Hubei China; 3 Division of Gastroenterology and Hepatology, Department of Medicine, University of North Carolina, Chapel Hill, NC 27516 USA; 4 Saint Louis University Liver Center, Saint Louis University School of Medicine, St. Louis, MO 63104 USA

**Keywords:** Hepatitis C virus, Multiple displacement amplification, Capture sequencing, Duplex-specific nuclease, Direct acting antivirals

## Abstract

**Background:**

Viral relapse is a major concern in hepatitis C virus (HCV) antiviral therapy. Currently, there are no satisfactory methods to predict viral relapse, especially in the era of direct acting antivirals in which the virus often quickly becomes undetectable using PCR-based approaches that focus on a small viral region. Next-generation sequencing (NGS) provides an alternative option for viral detection in a genome-wide manner. However, owing to the overwhelming dominance of human genetic content in clinical specimens, direct detection of HCV by NGS has a low sensitivity and hence viral enrichment is required.

**Methods:**

Based on template-dependent multiple displacement amplification (tdMDA), an improved method for whole genome amplification (Wang et al., 2017. Biotechniques 63, 21–27), we evaluated two strategies to enhance the sensitivity of NGS-based HCV detection: duplex-specific nuclease (DSN)-mediated depletion of human sequences and HCV probe-based capture sequencing.

**Results:**

In DSN-mediated depletion, human sequences were significantly reduced in the two HCV serum samples tested, 65.3% → 55.6% → 33.7% (#4727) and 68.6% → 56% → 21% (#4970), respectively for no normalization, self- and driver-applied normalization. However, this approach was associated with a loss of HCV sequences perhaps due to its micro-homology with the human genome. In capture sequencing, HCV-mapped sequencing reads occupied 96.8% (#4727) and 22.14% (#4970) in NGS data, equivalent to 1936x and 7380x enrichment, respectively. Capture sequencing was then applied to ten serum samples collected at the end of HCV antiviral therapy. Interestingly, the number of HCV-mapped reads was significantly higher in relapsed patients (*n* = 5) than those from patients with sustained virological response (SVR) (*n* = 5), 102.4 ± 72.3 vs. 2.6 ± 0.55, *p* = 0.014.

**Conclusions:**

Our data provides concept evidence for a highly sensitive HCV detection by capture sequencing. The abundance difference of HCV sequencing reads at the end of HCV antiviral therapy could be applied to predict treatment outcomes.

## Background

The development of direct acting antivirals (DAAs) is a milestone in antiviral therapy for hepatitis C virus (HCV) infection. Given the high rate of sustained virological response (SVR) among patients receiving DAA regimens, HCV infection is now claimed to be a curable disease [1]. In reality, however, there remains a small portion of patients, around 5%, who have the virus relapsed after treatment cessation [2]. HCV is circulating as a heterogenous population, sometime termed viral quasispecies [3]. In such a population, relapse is often accompanied with the enrichment of resistance-associated mutations that may foster the emergence of new drug-resistant viral strains [4]. Re-treatment with DAAs among relapsed patients confers a reduced SVR rate [5]. Viral relapse is thus a major concern in the era of DAAs. As HCV completes its life cycle solely in cytoplasm and has no evidence of cellular integration [6], its survival depends on continuous replication. Consequently, patients at the time of HCV relapse should maintain a low-level viral replication that is beyond the detection limit of current PCR-based methods [7]. In this setting, an alternative option is next-generation sequencing (NGS) that may detect the virus in a genome-wide manner. A drawback of using NGS for viral detection is its low sensitivity owing to the overwhelming dominance of human and commensal microbial genomes in clinical specimens [8]. Using specially designed primers, our lab previously developed template-dependent multiple displacement amplification (tdMDA) that eliminates primer-mediated artifacts [9]. Based on this improved whole genome amplification method, the current study attempted to increase the sensitivity of NGS through two enrichment strategies, depletion of human sequences or target capture by probe-based hybridization. The optimized method was then applied to clinical serum samples to explore the feasibility in detecting extremely low viral titers, thereby providing an on-treatment tool in predicting the outcomes of HCV antiviral therapy.

## Methods

### Patient samples

A total of 12 patient serum samples were included in the current study. Of the 12, two samples from our previous study, #4727 and #4970 (both genotype 1a), were collected prior to antiviral therapy and had viral loads respectively at 8.72 × 10^6^ and 5.24 × 10^5^ copies/mL as quantified by Roche Amplicor HCV Monitor (v2.0) [10]. These two samples were used for methodological optimization. The other ten samples were collected at the end of treatment (EOT) from the patients receiving either pegIFNα2a/ribavirin (*n* = 3) or a DAA regimen (boceprevir and telaprevir) (*n* = 7). All patients were infected with HCV genotype 1a. The samples were respectively collected from a completed clinical study (pegIFNα2a) and Hepatitis C Therapeutic Registry and Research Network (TARGET) (DAA regimen) ClinicalTrials.gov Identifier: NCT01474811 [10, 11]. Of the ten patients, five achieved SVR (2 with IFN and 3 with DAA) and the other five patients had virus relapses (1 with IFN and 4 with DAA). The study protocol was reviewed and approved by the Saint Louis University Institutional Review Board (IRB protocol 10,592).

### RNA extraction, reverse transcription (RT), tdMDA, and Illumina sequencing

RT-tdMDA was conducted as previously described [9]. In brief, total RNA was extracted from 140 μL of serum (samples #4727 and #4970) and eluted into 60 μL Tris buffer (pH 8.5) using the QIAamp Viral RNA Mini kit (Qiagen, Valencia, CA). An aliquot of 10.6 μL of extracted RNA was mixed with 9.4 μL RT matrix consisting of 1x SuperScript III buffer, 10 mM DTT, 80 μM of exonuclease-resistant random pentamer primers with the 5′ end blocked by C18 spacer [9], 2 mM dNTPs (Epicentre), 20 U (0.5 μL) of RNaseOUT Recombinant Ribonuclease Inhibitor (Invitrogen), and 200 U of SuperScript III reverse transcriptase (Life technologies). After incubation at 37 °C for 30 min and 50 °C for 30 min, the reaction was inactivated at 70 °C for 15 min. An aliquot of 4 μL of RT was used for tdMDA in 40-μL reaction consisting of 1x phi29 DNA polymerase buffer, 1 mM of dNTPs, 80 μM of random pentamer primers as used in the RT, and 20 units of phi29 DNA polymerase (New England Biolabs, Ipswich, MA). The reaction was incubated at 28 °C for 14 h and then terminated by being heated at 65 °C for 15 min. After purification with the QIAamp DNA mini kit (Qiagen), RT-tdMDA product was subjected to library construction with the Nextera XT DNA Sample Preparation kit (Illumina, San Diego, CA) and sequenced on the Illumina NextSeq 500 platform (1 × 250-nt single reads and mid-output) at MOgene (St. Louis, MO).

### Enrichment of HCV detection via the depletion of human sequences

This approach utilized duplex-specific nuclease that showed optimal activity at high temperatures with a strong preference for cleavage of double-stranded DNA in comparison to single-strand DNA [12]. Mixed human genomic DNA (male and female) (Promega, Madison, WI) was sheared into ~ 150 bp fragments using the Covaris microTUBE device, followed by purification with AMPure XP beads (Beckman Coulter, Indianapolis, IN), first at 0.8x for removing large-size fragments and then at 2.5x for the harvest (Supplementary Figure 1). Next, using an optimized protocol (Supplementary Figure 2), 300 ng of purified RT-tdMDA product was fragmented through an 8-min heating at 98 °C and then mixed with 1200 ng (1:2 or 1:4) of sheared human genomic DNA (the driver) in a 20-μL reaction containing 1x hybridization buffer [50 mM HEPES (pH 7.5) and 0.5 M NaCl]. After being heated at 98 °C for 3 min, the reaction was incubated at 68 °C for 4 h. At the end of the incubation, 8 μL of pre-warmed solution, including 2 μL of DSN (1 U/μL) (Evrogen via Axxora, Farmingdale, NY) and 2.8 μL of 10x DSN buffer, was added for an additional 30-min incubation. The reaction was terminated by adding 28 μL (1:1) of 2x DSN stop solution and then subjected to two-step purification, first by the QIAquick PCR Purification Kit (Qiagen) and then AMPure XP beads at 0.55x that was determined by titration for the size exclusion of < 300 bp fragments (Supplementary Figure 3). The entire purified product was used for 4-h tdMDA. These experiments were repeated multiple times and the efficiency of enrichment was initially estimated using an in-house PCR based on HCV 5’UTR (30 cycles of single round) [13]. Selective tdMDA product was then processed into Illumina sequencing as described above.

### Enrichment of HCV detection via capture sequencing

This strategy was applied to samples #4727 and #4970. First, 384 full-length HCV genotype 1a genomes were extracted from the Los Alamos HCV database to generate a consensus sequence [14], which was 9508 nt in length without 120-nt poly(U)/C tract (Supplementary File 1). The 2x HCV titling probes (each 120 nt with 60 nt overlap) were designed using an in-house script and synthesized at Integrated DNA Technologies (IDT) (Coralville, IA). After regular library preparation from RT-tdMDA product, the library was hybridized with probes using the xGen Hybridization and Wash Kit (IDT), followed by PCR amplification, quantitation, and finally the sequencing run as described above.

### Data analysis

Raw sequence reads in fastq format from samples were filtered in PRINSEQ (v0.20) for quality control, including read length ≥ 70 nt, mean read quality score ≥ 25, low complexity with DUST score ≤ 7, ambiguous bases ≤1%, and all types of duplicates [15]. To reduce non-specific HCV mapping, quality reads were first subtracted by human sequences [The National Center for Biotechnology Information (NCBI) GRCh38 build] [16] using the Bowtie 2 mapper [17]. Remaining reads were then mapped onto the HCV genotype 1a probe sequence. Read alignment was converted, sorted, and indexed into BAM file in SAMtools and was viewed in BamView [18, 19]. When necessary, HCV consensus sequences were called using two-step procedure as described in our previous study [20]. HCV consensus sequences, together with HCV sequences generated through cloning and Sanger sequencing from the same samples [10], were used for the construction of the phylogenetic tree in MEGA (Molecular Evolutionary Genetics Analysis) [21].

### Analysis of micro-homology between HCV 1a consensus sequence and the human genome

Micro-homology may affect the efficiency of the enrichment method, which can be either DSN-mediated depletion of human genomic sequences or HCV capture sequencing. To analyze its potential roles, HCV 1a probe sequence was split into 60-nt fragments with 30-bp overlaps using pyfasta [22]. These sequences were searched for micro-homology against the human genome (GRCh38 build) by blastn at a word size of 6. Based on the blastn output, human genomics sequences were extracted using HOMER with additional steps for the removal of duplicates [23]. Non-redundant human genomics sequences were calculated for the melting temperatures (Tm) using the nearest neighbor method [24].

### Detection of HCV at the end of antiviral therapy by capture sequencing

After comparing the sensitivity between the two enrichment options, capture sequencing was selected to detect HCV from the 10 serum samples collected at EOT. All serum samples were tested for HCV using in-house RT-PCR protocol (total 70 cycles) based on 5′ untranslated region (5’UTR) as described previously [11]. RT-tdMDA from a healthy donor serum and water were included as the negative control. The experimental procedure for capture sequencing was the same as what was described for samples #4727 and #4970.

### Statistical analysis

Student’s t-test. Data were expressed as the mean ± SD (standard deviation), and *p* < 0.05 was considered statistically significant.

### Data availability

Raw sequence data in fastq format from the current study were deposited in the NCBI Sequence Read Archive (SRA) under BioProject ID: PRJNA545854.

## Results

### Enrichment comparison: DSN-mediated depletion vs. capture sequencing

**Fig. 1:**
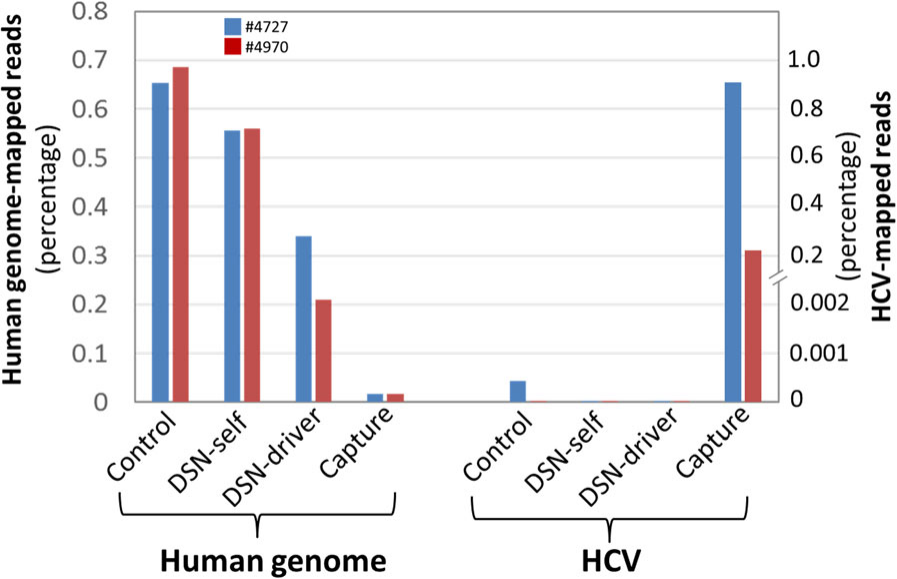
HCV enrichment by DSN-mediated normalization and capture sequencing. Reads for human genome was mapped using Bowtie 2 onto NCBI GRCh38 build and calculated as percentages (left Y-axis) while HCV was mapped using 184 reference sequences from the Los Alamos HCV database (right Y-axis)

Following RT-tdMDA, samples #4727 and #4970 were subjected to Illumina sequencing under three options, regular library preparation, DSN-mediate depletion of human genomic sequences prior to library construction, and capture hybridization. Under regular sequencing, only 585 (0.05%) and 40 (0.003%) reads were mapped onto the HCV genotype 1a consensus sequence for samples #4727 and #4970 respectively (Supplementary Table 1). Both samples had reads from the human genome dominate the data, 65.3% for #4727 and 68.6% for #4970. In DSN-based normalization, HCV PCR didn’t indicate an enrichment effect (data not shown). This was confirmed by subsequent Illumina sequencing. While human reads were reduced to 55.6 and 56% in self-normalization and 33.7 and 21% with the use of the driver for samples #4727 and #4970 respectively, self- and driver-based normalization were associated with the loss of HCV-mapped reads, 585 → 19 → 3 for #4727 and 40 → 4 → 1 for #4970 (Fig. 1). In contrast, capture sequencing had 96.8 and 22.14% reads mapped onto the HCV genotype 1a consensus sequence, which was equivalent to approximately 1936x and 7380x enrichment for #4727 and #4970 respectively (Fig. 1).

### Micro-homology may contribute the loss of HCV sequences in DSN-mediated sequencing

**Fig. 2:**
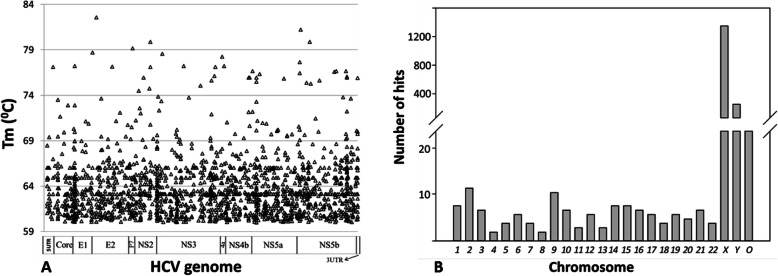
Micro-homology between human genome and HCV genotype 1a consensus sequence. Micro-homologous regions were shown along with the entire HCV genome with corresponding Tm values (**a**). Distribution of micro-homologous regions across human chromosomes with Tm values higher than 60 °C

Two factors were speculated to be responsible for the loss of HCV-mapped reads in DSN-mediated normalization. First, DSN activity appeared to be harmful for phi29 DNA polymerase. When DSN was inactivated through the procedure recommended by the manufacturer, tdMDA after purification with AMPure XP beads was inefficient (data not shown). For a complete removal of DSN, an additional purification step using Qiagen spin columns was required after DSN digestion. More purification steps may result in a loss of low-abundant template, such as HCV sequences. Another factor was the micro-homology between HCV and the human genome. At ≥95% similarity, the HCV genotype 1a consensus sequence baited 1784 unique regions, ranging from 11 to 25 nt, on the human genome with Tm higher than 60 °C. While these regions were scattered across the entire HCV genome (Fig. 2a), it was interesting to note that there was uneven distribution among chromosomes with 78% of hits contributed by chromosome X (Fig. 2b). In the existence of excessive human DNA, digestion of HCV sequences owing to the micro-homology appears to be a more plausible explanation.

### Authentic recovery of HCV genomes by capture sequencing

**Fig. 3:**
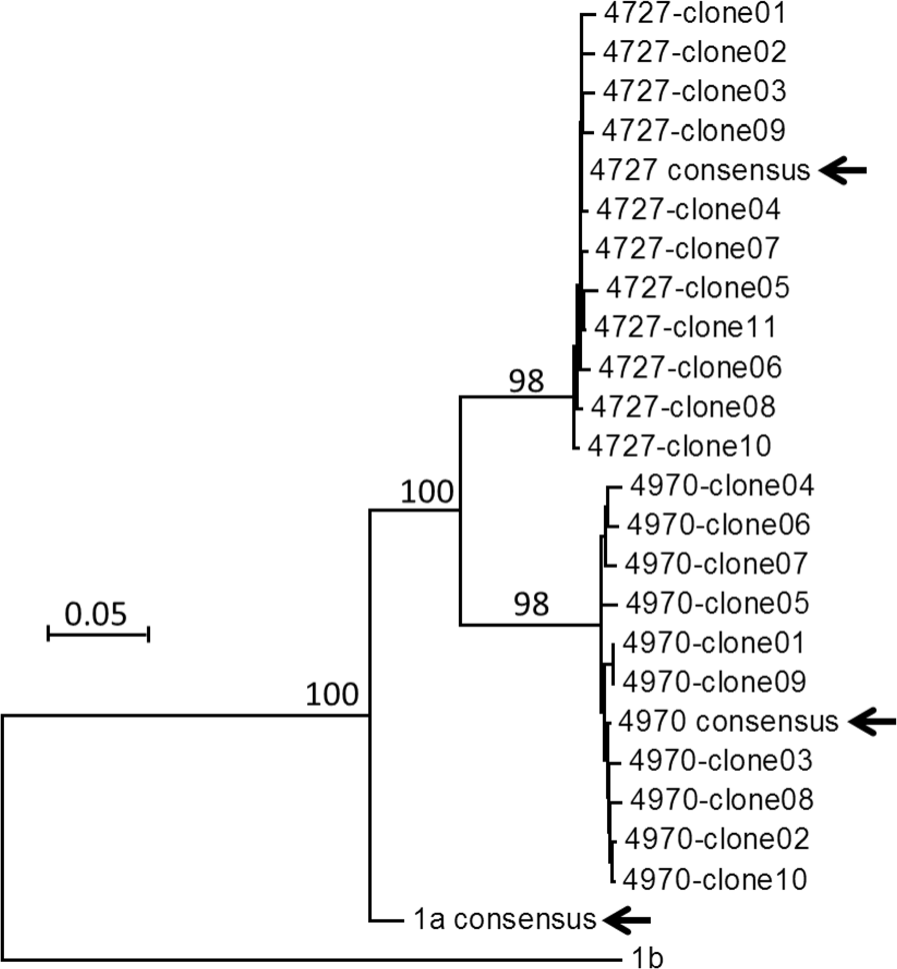
The Neighbor-Joining tree of 1340-bp HCV sequences. The tree was constructed using the Tamura 3-parameter method with bootstrap values (1000 replicates) shown on major branches. Arrows indicated the consensus sequences called from capture sequencing. Also indicated was the HCV genotype 1a consensus sequence that was used for probe deign in capture sequencing

Samples #4727 and #4970 were studied for viral quasispecies through cloning and Sanger sequencing of the 1380-bp amplicon across HCV Core, E1 and E2 regions [8]. After the removal of primer sequences, the 1340-bp consensus sequence derived from multiple clones shared 90% (#4727) and 88% (#4970) nucleotide similarity with the HCV genotype 1a consensus sequence that was used for probe design. However, the consensus sequences from cloning and capture sequencing had much higher nucleotide similarity, 99.4 and 99.25% for samples #4727 and #4970 respectively. Accordingly, the consensus sequences called from capture sequencing were phylogenetically well-clustered with cloned HCV sequences in both samples (Fig. 3). In addition, capture sequencing gave a full coverage across the entire HCV genome without any gaps (data not shown). Thus, probe design at the level of HCV subtypes could tolerate sequence divergence among HCV strains to ensure accurate recovery of HCV genomes.

### Detection of HCV at the end of antiviral therapy

**Fig. 4:**
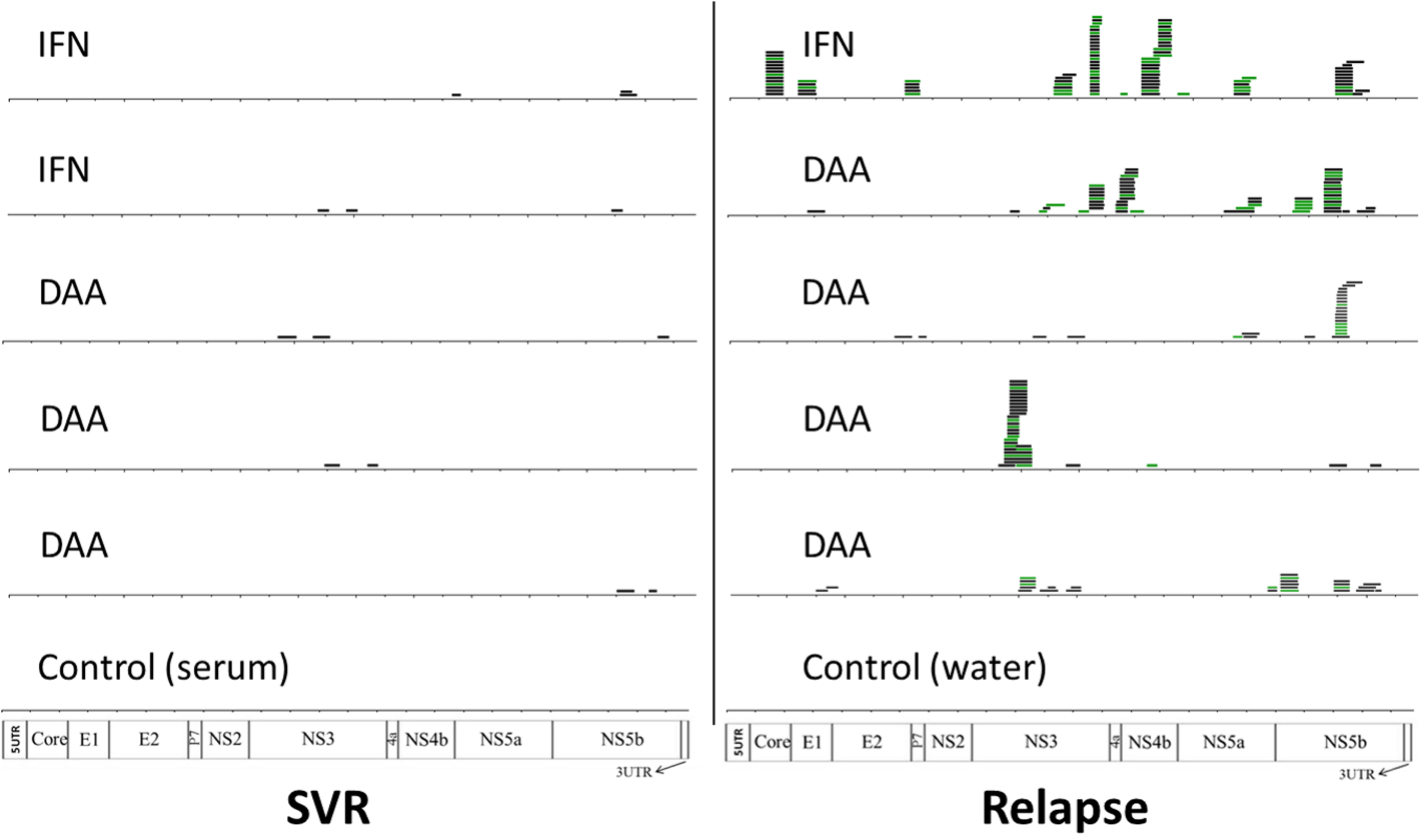
Alignment of HCV-mapped reads on the HCV genotype 1a consensus sequence. Patients were grouped based on treatment outcomes (SVR or relapse) and indicated with treatment regimens (IFN or DAA)

The experimental protocol for HCV capture sequencing was applied to the 10 serum samples collected at the end of antiviral therapy. These samples tested negative using a commercial kit (COBAS AmpliPrep/COBAS TaqMan HCV Test, v2.0) and in-house RT-nested PCR (data not shown). Using the same probes from HCV genotype 1a consensus sequence, capture sequencing detected 3, 3, 3, 2, and 2 reads in five patients with SVR, which was a sharp contrast to the five patients with viral relapse that had 208, 148, 48, 56, and 52 reads mapped (Fig. 4) (Supplementary Table 1). No HCV-mapped reads were found in two negative controls (Fig. 4). Quantitatively, SVR patients had an average 2.6 ± 0.55 reads in comparison to 102.4 ± 72.3 from relapsed patients (*p* = 0.014 two-tailed Student t test). And the similar result was obtained after the normalization to the mean number of total reads among these patients (111.4 ± 81.5 vs. 2.64 ± 0.27, *p* = 0.017). Read mapping showed the lack of a consistent pattern with the reads scattered across the HCV genome (Fig. 4). Finally, the average rate of duplicate reads among 10 samples was 29.8 ± 7.8%. Based on this rate, the estimated library size was 1.01 ± 0.2 million of reads under a Poisson distribution [25]. Therefore, capture sequencing in these kinds of samples have small library sizes that could be easily achieved with current NGS service.

## Discussion

Based on tdMDA, the current study has explored two options to enhance the sensitivity of clinical HCV sequencing from patient serum samples. DSN has a good activity at high temperature ranging from 60 °C to 80 °C [12]. At these temperatures, abundant DNA species after denaturation in a sample have more chances to form dsDNA that are digested while rare species are intact in the form of ssDNA [12]. Consequently, a normalization among DNA species is achieved. This unique characteristic of DSN is widely used for NGS library normalization and for the depletion of individual genes [26–29]. Our study confirmed a DSN-mediated normalization in RT-tdMDA product with or without the use of the sheared human genomic DNA (the driver). As illustrated by the ratios of human sequences in NGS data (Fig. 1), inclusion of the driver appears to be more effective than self-normalization (no driver). However, in either self- or driver-mediated normalization, HCV sequences were digested simultaneously, potentially owing to the micro-homology between HCV and the human genome (Fig. 2). In contrast, capture sequencing significantly enriched the recovery of HCV sequences and had no notable off-target effect. Sample #4727 has a HCV on-target rate of 96.8%, which is 4.37 times higher than that from sample #4970 (22.14%) (Fig. 1). Because a pivotal step in capture sequencing is probe-based hybridization, its efficiency is affected by the target’s abundance and sequence divergence from the probe. In our study, both samples #4727 and #4970 belongs to HCV genotype 1a and have similar genetic distance to the HCV genotype 1a consensus sequence (probe) (Fig. 3). Therefore, difference in HCV on-target rate could be attributed to viral titer that was 16.6 times higher in sample #4727 than #4970. Indeed, sample #4970 had a greater enrichment efficiency (7380x) in comparison to #4727 (1936x), illustrating the power of capture sequencing.

Our data is consistent with recent reports that use similar strategy for HCV sequencing [30, 31]. The enrichment of our samples is even higher than those reported [30, 31]. Besides the complete elimination of primer-originated artifacts, tdMDA uses phi29 DNA polymerase that favors the amplification of long templates [32]. The utilization of tdMDA for whole transcriptome amplification would intrinsically enrich HCV sequences in comparison to direct library preparation of serum RNA. Finally, by comparing the sequences from amplicon-based cloning/Sanger sequencing, the study also provides evidence for the sequence authenticity using capture probes designed at the level of HCV subtype (1a) consensus sequence (Fig. 3).

After the application of capture sequencing to serum samples collected at EOT, there was significant difference between patients with SVR and viral relapse in terms of the number of HCV reads detected (Fig. 4). The reads showed a scattered pattern without a full coverage across HCV genome, supporting extraordinarily low viral loads in blood. Indeed, these samples were all negative by using RT-PCR based on HCV 5′ UTR. In our experience, a 70-cycle of nested RT-PCR is even more sensitive than Amplicor HCV Monitor (v2.0) that has a detection limit at 50 IU/mL, equivalent to 45 copies/mL [13, 33–35]. Using commercial kits, however, HCV is reported to be detectable and even quantifiable at EOT in a small percentage of patients who achieve SVR [36, 37]. Regardless of drug regimens, HCV antiviral therapy requires certain duration in order to eradicate the virus, usually 48 weeks for IFN-based therapy or 8–12 weeks for DAAs. In view of evolutionary biology, such duration represents a bottleneck that fosters the generation and accumulation of deleterious mutations, so-called Miller’s ratchet [38–40], which eventually renders the loss of replicative fitness, thereby virus extinction. Our previous studies have documented the link between HCV mutation loads and the outcomes of IFN-based antiviral therapy [20, 41]. When deleterious mutations approach a level that prevents the rescue of replicative fitness after treatment cession, the virus goes extinct in spite of a positive HCV detection at EOT. At this point, it is necessary to discriminate quantitative and qualitative HCV genomic characteristics between SVR and relapse when detectable at EOT. The current study includes a small number of patients (5 SVR and 5 relapse), which represents a major limitation. in the current study. Further study with more patient samples is required for a solid clinical and evolutionary interpretation in terms of HCV detection by capture sequencing at EOT.

## Conclusions

In summary, DSN-mediated depletion of human sequences is an inefficient strategy for the enrichment of HCV sequences in NGS. In contrast, the current study provides preliminary evidence that tdMDA, in combination with HCV capture sequencing, could be used as a highly sensitive tool in detecting HCV. As predicted by evolutionary principles, tailoring treatment duration based on HCV detection at EOT could reduce and even eliminate the incidence of viral relapse. Given the high cost of medicines like DAAs, the method described here allows a cost-effective personalized management in HCV clinic.

## Supplementary information

**Additional file 1 **: **Figure S1.** Preparation of human genome baits. **Figure S2.** Fragmentation of RT-tdMDA product by heating. **Figure S3.** Titration of Ampure XP beads in DNA purification. **Table S1.** Mapping statistics of 20 samples. **Supplementary file 1.** HCV genotype 1a consensus sequence: 9508 bp.

## Data Availability

Raw sequence data from the current study were deposited in the NCBI Sequence Read Archive (SRA) under BioProject ID: PRJNA545854. The HCV genotype 1a consensus sequence was provided in the Supplementary information.

## Abbreviations

NGS: next-generation sequencing
tdMDA: Template-dependent multiple displacement amplification
HCV: Hepatitis C virus
DSN: Duplex-specific nuclease
DAA: Direct acting antivirals
SVR: Sustained virological response
EOT: End of treatment
TARGET: Hepatitis C Therapeutic Registry and Research Network
