# Supplementary material for: Genome-wide capture sequencing to detect hepatitis C virus at the end of antiviral therapy
Source: BMC Infect Dis. 2020 Aug 26;20:632. doi: 10.1186/s12879-020-05355-2 (PMC7448998; doi:10.1186/s12879-020-05355-2)
Supplement: Supplementary file 1 — Additional file 1 : Figure S1. Preparation of human genome baits. Figure S2. Fragmentation of RT-tdMDA product by heating. Figure S3. Titration of Ampure XP beads in DNA purification. Table S1. Mapping statistics of 20 samples. Supplementary file 1. HCV genotype 1a consensus sequence: 9508 bp. [file 12879_2020_5355_MOESM1_ESM.pdf]

**Peng et al. Genome-wide Capture Sequencing to Detect Hepatitis C Virus at the End of Antiviral Therapy**

# Supplementary material

**Supplementary Figure 1. Preparation of human genome baits.**

**Supplemental Figure 2. Fragmentation of RT-tdMDA product by heating.**

**Supplementary Figure 3. Titration of Ampure XP beads in DNA purification.**

**Supplementary Table 1. Mapping statistics of 20 samples.**

**Supplementary File 1. HCV genotype 1a consensus sequence: 9,508 bp**

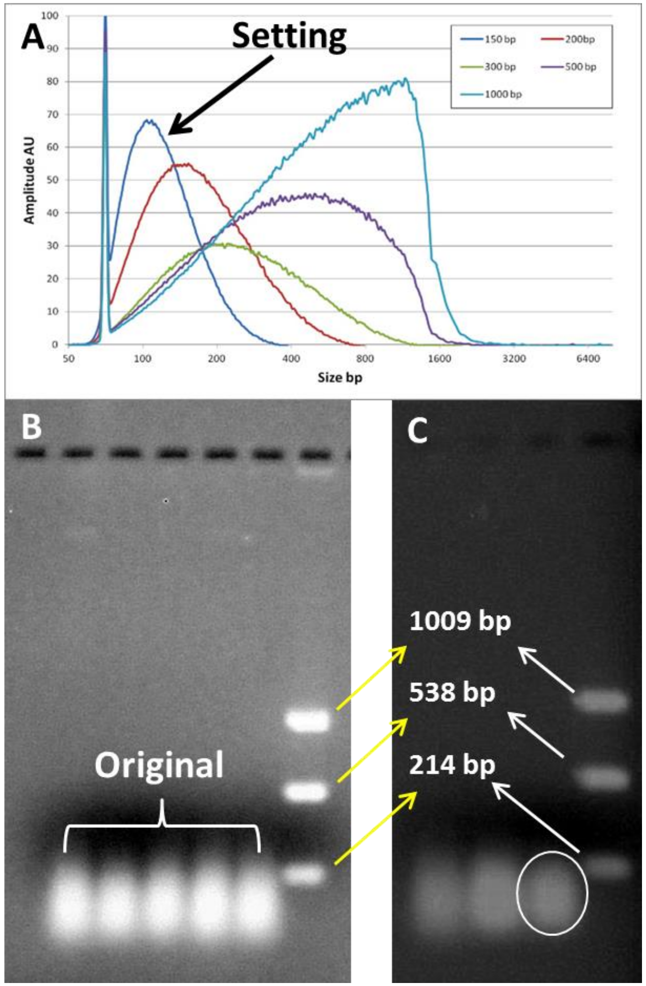


**Supplementary Figure 1. Preparation of human genome baits.** Mixed human genomic DNA (Promega) was sheared in Covaris under 100-bp setting (A). After similar titrations, the original sheared product (B) was purified using AMpure XP beads, first at 0.8x for excluding large size and then at 2.5x for the harvest (circled band in C).

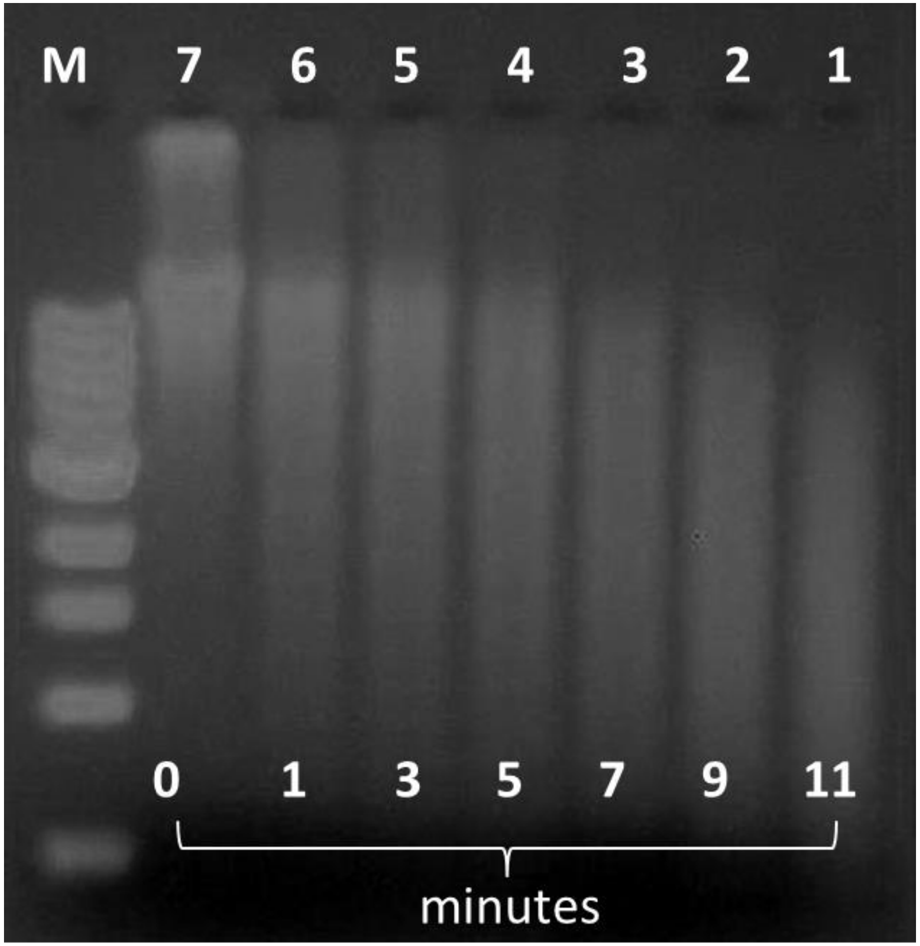


**Supplemental Figure 2. Fragmentation of RT-tdMDA product by heating.** Fragmentation of RT-tdMDA was achieved by heating with different incubation lengths. Eventually, a 9-minute incubation was chosen for fragmentation that generated the fragments within a range of 1 to 10 kb. M, 1 kb DNA ladder.

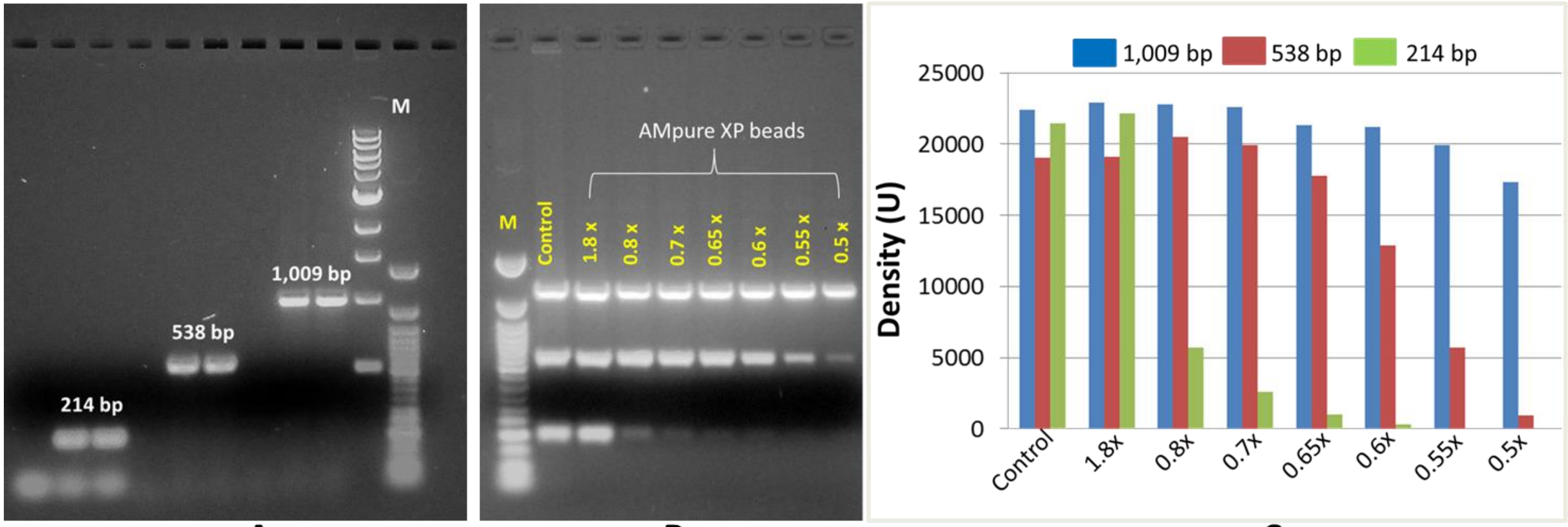


A B C

**Supplementary Figure 3. Titration of Ampure XP beads in DNA purification.** Three amplicons with different sizes were generated by PCR from a recombinant HCV plasmid (A). Amplicons, purified using Qiagen Spin column, were pooled for size exclusion under various rates of AMpure XP beads (B). Quantitation of gel bands was conducted using NIH ImageJ (C).

| No | Serum no | Antiviral therapy | | Time point | Enrichment | Total | Human | | HCV | | Other | |
|---|---|---|---|---|---|---|---|---|---|---|---|---|
| | | Regimen | Outcome | | | | Number | % | Number | % | Number | % |
| 1 | #4727 | No treatment | | Baseline | Capture | 1290018 | 20403 | 1.58% | 1248737 | 96.80% | 20878 | 1.62% |
| 2 | #4727 | | | | Regular | 1214237 | 792847 | 65.30% | 518 | 0.04% | 420872 | 34.66% |
| 3 | #4727 | | | | DSN-self | 1087403 | 604676 | 55.61% | 19 | 0.00% | 482708 | 44.39% |
| 4 | #4727 | | | | DSN-driver | 917965 | 309984 | 33.77% | 3 | 0.00% | 607978 | 66.23% |
| 5 | #4970 | | | | Capture | 954773 | 16442 | 1.72% | 211386 | 22.14% | 726945 | 76.14% |
| 6 | #4970 | | | | Regular | 1360962 | 933517 | 68.59% | 40 | 0.00% | 427405 | 31.40% |
| 7 | #4970 | | | | DSN-self | 707477 | 394630 | 55.78% | 4 | 0.00% | 312843 | 44.22% |
| 8 | #4970 | | | | DSN-driver | 504344 | 105662 | 20.95% | 1 | 0.00% | 398681 | 79.05% |
| 9 | #0777 | IFN | Relapse | EOT | Capture | 798647 | 11157 | 1.40% | 208 | 0.03% | 787282 | 98.58% |
| 10 | #0954 | DAA | | | Capture | 782602 | 19010 | 2.43% | 148 | 0.02% | 763444 | 97.55% |
| 11 | #0466 | DAA | | | Capture | 1337552 | 1074 | 0.08% | 48 | 0.00% | 1336430 | 99.92% |
| 12 | #2765 | DAA | | | Capture | 999625 | 3266 | 0.33% | 56 | 0.01% | 996303 | 99.67% |
| 13 | #2877 | DAA | | | Capture | 504255 | 105520 | 20.93% | 52 | 0.01% | 398683 | 79.06% |
| 14 | #3711 | IFN | SVR | | Capture | 872656 | 105590 | 12.10% | 3 | 0.00% | 767063 | 87.90% |
| 15 | #4756 | IFN | | | Capture | 884940 | 129034 | 14.58% | 3 | 0.00% | 755903 | 85.42% |
| 16 | #5617 | DAA | | | Capture | 1091724 | 20321 | 1.86% | 3 | 0.00% | 1071400 | 98.14% |
| 17 | #7811 | DAA | | | Capture | 698649 | 84757 | 12.13% | 2 | 0.00% | 613890 | 87.87% |
| 18 | #0221 | DAA | | | Capture | 672291 | 120749 | 17.96% | 2 | 0.00% | 551540 | 82.04% |
| 19 | Serum from a healthy blood donor | | | | Capture | 1119714 | 77402 | 6.91% | 0 | 0.00% | 1042312 | 93.09% |
| 20 | Water | | | | Capture | 634926 | 12697 | 0.02% | 0 | 0.00% | 622229 | 98.00% |

**Supplementary Table 1.** Mapping statistics of 20 samples. The number of total reads was counted after the quality control. Reads for human genome were mapped using Bowtie 2 onto NCBI GRCh38 build and calculated as percentages while HCV was mapped using 184 reference sequences from the Los Alamos HCV database. Reads in the category of “Other” were mapped to microbe genomes from human commensal viruses and bacteria or from reagents from kits and experimental pipelines. Annotation of this category of reads was detailed in our recent studies (Transfusion 2019; 59:3177-85; Arch Virol. 2020; 165:127-35). EOT, end of treatment; SVR, sustained virological response.

```
>HCV_genotype_1a_consensus_sequence_9508bp
GCCAGCCCCCTGATGGGGGCGACACTCCACCATGAATCACTCCCCTGTGAGGAACTATTGTCTTCACGCAGAAAGCGTCTAGC
CATGGCGTTAGTATGAGTGTCGTGCAGCCTCCAGGACCCCCCCTCCCGGGAGAGCCATAGTGGTCTGCGGAACCGGTGAGTAC
ACCGGAATTGCCAGGACGACCGGGTCCTTTCTTGGATAAACCCGCTCAATGCCTGGAGATTTGGGCGTGCCCCCGCAAGACTG
CTAGCCGAGTAGTGTTGGGTCGCGAAAGGCCTTGTGGTACTGCCTGATAGGGTGCTTGCGAGTGCCCCGGGAGGTCTCGTAGA
CCGTGCACCATGAGCACGAATCCTAAACCTCAAAGAAAAACCAAACGTAACACCAACCGTCGCCCACAGGACGTCAAGTTCCC
GGGTGGCGGTCAGATCGTTGGTGGAGTTTACTTGTTGCCGCGCAGGGGCCCTAGATTGGGTGTGCGCGCGACGAGGAAGACTT
CCGAGCGGTCGCAACCTCGAGGTAGACGTCAGCCTATCCCCAAGGCGCGTCGGCCCGAGGGCAGGACCTGGGCTCAGCCCGGG
TACCCTTGGCCCCTCTATGGCAATGAGGGCTGCGGGTGGGCGGGATGGCTCCTGTCTCCCCGTGGCTCTCGGCCTAGCTGGGG
CCCCACAGACCCCCGGCGTAGGTCGCGCAATTTGGGTAAGGTCATCGATACCCTCACGTGCGGCTTCGCCGACCTCATGGGGT
ACATACCGCTCGTCGGCGCCCCTCTTGGAGGCGCTGCCAGGGCCCTGGCGCATGGCGTCCGGGTTCTGGAAGACGGCGTGAAC
TATGCAACAGGGAACCTTCCTGGTTGCTCTTTCTCTATCTTCCTTCTGGCCCTGCTCTCTTGCCTGACTGTGCCCGCTTCAGC
CTACCAAGTGCGCAACTCCACGGGGCTTTACCATGTCACCAATGATTGCCCTAACTCGAGTATTGTGTACGAGGCGGCCGATG
CCATCCTGCACACTCCGGGGTGTGTCCCTTGCGTTCGCGAGGGTAACGCCTCGAGGTGTTGGGTGGCGGTGACCCCCACGGTG
GCCACCAGGGACGGCAAACTCCCCACAACGCAGCTTCGACGTCACATCGATCTGCTTGTCGGGAGCGCCACCCTCTGCTCGGC
CCTCTACGTGGGGGACCTGTGCGGGTCTGTCTTTCTTGTCGGTCAACTGTTCACCTTCTCTCCCAGGCGCCACTGGACGACGC
AAGACTGCAATTGTTCTATCTATCCCGGCCATATAACGGGTCACCGCATGGCATGGGATATGATGATGAACTGGTCCCCTACG
ACGGCGTTGGTAGTAGCTCAGCTGCTCCGGATCCCACAAGCCATCTTGGACATGATCGCTGGTGCTCACTGGGGAGTCCTGGC
GGGCATAGCGTATTTCTCCATGGTGGGGAACTGGGCGAAGGTCCTGGTAGTGCTGCTGCTATTTGCCGGCGTCGACGCGGAAA
CCCACGTCACCGGGGGAAGTGCCGCCCGCACCGCGTCTGGACTTGCCAGTCTCTTCACACCAGGCGCCAAGCAGAACATCCAG
CTGATCAACACCAACGGCAGTTGGCACATCAATAGCACGGCCTTGAACTGCAATGACAGCCTTGACACCGGCTGGATAGCAGG
GCTTTTCTATTACCACAAATTCAACTCTTCAGGCTGTCCCGAGAGGTTGGCCAGCTGCCGACCCCTTACCGATTTTGACCAGG
GCTGGGGCCCTATCAGTTATGCCAACGGAAGCGGCCCCGACCAACGCCCCTACTGCTGGCACTACCCCCCAAAACCTTGTGGT
ATTGTGCCCGCAAAGAGCGTGTGTGGCCCGGTATATTGCTTCACTCCCAGCCCCGTGGTGGTGGGAACGACCGACAGGTCGGG
CGCGCCTACCTACAACTGGGGTGAAAATGATACGGACGTCTTCGTCCTTAACAACACCAGGCCACCGCTGGGCAATTGGTTCG
GTTGCACCTGGATGAACTCAACTGGATTCACCAAAGTGTGCGGAGCGCCCCCTTGTGTCATCGGAGGGGTGGGCAACAACACC
TTGCACTGCCCCACTGATTGTTTCCGCAAGCATCCGGAAGCCACATACTCTCGGTGCGGCTCCGGTCCCTGGATCACACCCAG
GTGCCTGGTCCACTACCCGTATAGGCTTTGGCATTATCCTTGTACCATCAACTACACCATATTCAAAGTCAGGATGTACGTGG
GAGGGGTCGAGCACAGGCTGGAAGCTGCCTGCAACTGGACGCGGGGCGAGCGTTGCGATCTGGAAGACAGGGACAGGTCCGAG
CTCAGCCCGTTGCTGCTGTCCACCACACAGTGGCAGGTCCTTCCGTGTTCCTTCACGACCCTGCCAGCCTTGTCCACCGGCCT
CATCCACCTCCACCAGAACATCGTGGACGTGCAGTACTTGTACGGGGTGGGGTCAAGCATCGCGTCCTGGGCCATCAAGTGGG
AGTACGTCGTTCTCCTGTTCCTTCTGCTTGCAGACGCGCGCGTCTGCTCCTGCTTGTGGATGATGTTACTCATATCCCAAGCG
GAGGCGGCTTTGGAGAACCTCGTAATACTCAATGCAGCATCCCTGGCCGGGACGCACGGTCTTGTGTCCTTCCTCGTGTTCTT
CTGCTTTGCATGGTATCTGAAGGGTAGGTGGGTGCCCGGAGCGGCCTACGCCCTCTACGGGATGTGGCCTCTCCTCCTGCTCC
TGTTGGCGTTGCCCCAGCGGGCATACGCGCTGGACACGGAGGTGGCCGCGTCGTGTGGCGGCGTTGTTCTTGTCGGGTTAATG
GCGCTGACTCTGTCACCATATTACAAGCGCTATATCAGCTGGTGCTTATGGTGGCTTCAGTATTTTCTGACCAGAGTAGAAGC
GCAACTGCACGTGTGGGTTCCCCCCCTCAACGTCCGAGGGGGGCGCGACGCCGTCATCTTACTCATGTGTGTTGTACACCCGA
CTTTGGTATTTGACATCACCAAACTGCTGCTGGCCGTCTTCGGACCCCTTTGGATTCTTCAAGCCAGTTTGCTTAAAGTGCCC
TACTTCGTGCGCGTTCAAGGCCTTCTCCGGATCTGCGCGCTAGCGCGGAAGATGGCCGGAGGCCATTACGTGCAAATGGCCAT
CATCAAGTTAGGGGCGCTTACTGGCACCTATGTTTATAACCATCTCACTCCTCTTCGGGACTGGGCGCACAACGGCCTGCGAG
ATCTGGCCGTGGCTGTAGAGCCAGTCGTCTTCTCCCAAATGGAGACCAAGCTCATCACGTGGGGGGCAGACACCGCCGCGTGC
GGTGACATCATCAACGGCTTGCCCGTCTCCGCCCGTAGGGGCCGGGAGATACTGCTCGGACCAGCCGATGGAATGGTCTCCAA
GGGGTGGAGGTTGCTGGCGCCCATCACGGCGTACGCCCAGCAGACAAGGGGCCTCCTAGGGTGCATAATCACCAGCCTGACTG
GCCGGGACAAAAACCAAGTGGAGGGTGAGGTCCAGATTGTGTCAACTGCTGCCCAAACTTTCCTGGCAACGTGCATCAATGGG
GTATGCTGGACTGTCTACCACGGGGCCGGAACGAGGACCATCGCATCACCCAAGGGTCCTGTTATCCAGATGTATACCAATGT
AGACCAAGACCTTGTGGGCTGGCCCGCTCCTCAAGGTGCCCGCTCATTGACACCCTGCACCTGCGGCTCCTCGGACCTTTACC
TGGTCACGAGGCACGCCGATGTCATTCCCGTGCGCCGGCGGGGTGATAGCAGGGGCAGCCTGCTTTCGCCCCGGCCCATTTCC
TACTTGAAAGGCTCCTCGGGGGGTCCGCTGTTGTGCCCCGCGGACACGCCGTAGGCATATTCAGGGCCGCGGTGTGCACCCG
TGGAGTGGCTAAGGCGGTGGACTTTATCCCTGTGGAGAACCTAGAGACAACCATGAGGTCCCCGGTGTTCACGGACAACTCCT
CTCCACCAGCAGTGCCCCAGAGCTTCCAGGTGGCCCACCTGCATGCTCCCACCGGCAGCGGTAAGAGCACCAAGGTCCCGGCT
GCATACGCAGCTCAGGGCTACAAGGTGCTAGTGCTCAACCCCTCTGTTGCTGCAACACTGGGCTTTGGTGCTTACATGTCCAA
GGCCCATGGGATCGATCCTAACATCAGGACCGGGGTGAGAACAATTACCACTGGCAGCCCCATCACGTACTCCACCTACGGCA
AGTTCCTTGCCGACGGCGGGTGCTCAGGGGGTGCTTATGACATAATAATTTGTGACGAGTGCCACTCCACGGATGCCACATCC
ATCTTGGGCATCGGCACTGTCCTTGACCAAGCAGAGACTGCGGGGGCGAGACTGGTTGTGCTCGCCACCGCTACCCCTCCGGG
CTCCGTCACTGTGCCCCATCCTAACATCGAGGAGGTTGCTCTGTCCACCACCGGAGAGATCCCTTTTTACGGCAAGGCTATCC
CCCTCGAGGTAATCAAGGGGGGGAGACATCTCATCTTCTGTCACTCAAAGAAGAAGTGCGACGAGCTCGCCGCAAAGCTGGTC
GCATTGGGCATCAATGCCGTGGCCTACTACCGCGGTCTTGACGTGTCTGTCATCCCGACCAGCGGCGATGTTGTCGTCGTGGC
AACTGATGCTCTCATGACCGGCTTTACCGGCGACTTCGACTCGGTGATAGACTGCAACACGTGTGTCACCCAGACAGTCGATT
TCAGCCTTGACCCTACCTTCACCATTGAGACAACCACGCTTCCCCAGGATGCTGTCTCCCGCACTCAACGTCGGGGCAGGACT
```

GGCAGGGGGAAGCCAGGCATCTACAGATTTGTGGCACCGGGGGAGCGCCCCTCCGGCATGTTCGACTCGTCCGTCCTCTGTGA
GTGCTATGACGCGGGCTGTGCTTGGTATGAGCTCACGCCCGCCGAGACTACAGTTAGGCTACGAGCGTACATGAACACCCCGG
GGCTTCCCGTGTGCCAGGACCATCTTGAATTTTGGGAGGGCGTCTTTACGGGCCTCACCCATATAGATGCCCACTTCCTATCC
CAGACAAAGCAGAGTGGGGAGAACTTTCCTTACCTGGTAGCGTACCAAGCCACCGTGTGCGCTAGGGCTCAAGCCCCTCCCCC
ATCGTGGGACCAGATGTGGAAGTGTTTGATCCGCCTCAAGCCCACCCTCCATGGGCCAACACCTCTGCTATACAGACTGGGCG
CTGTTCAGAATGAAGTCACCCTGACGCACCCAATCACCAAATACATCATGACATGCATGTCGGCTGACCTGGAGGTCGTCACG
AGCACCTGGGTGCTCGTTGGCGGCGTCCTGGCTGCTTTGGCCGCGTATTGCCTGTCAACAGGCTGCGTGGTCATAGTGGGCAG
GATTGTCTTGTCCGGGAAGCCGGCAATCATACCTGACAGGGAAGTCCTCTACCGGGAGTTCGATGAGATGGAAGAGTGCTCTC
AGCACTTACCGTACATCGAGCAAGGGATGATGCTCGCCGAGCAGTTCAAGCAGAAGGCCCTCGGCCTCCTGCAGACCGCGTCC
CGCCAGGCAGAGGTCATCGCCCCTGCTGTCCAGACCAACTGGCAAAAACTCGAGGCCTTCTGGGCGAAGCATATGTGGAACTT
CATCAGTGGGATACAATACTTGGCGGGCCTGTCAACGTTGCCTGGTAACCCCGCCATTGCTTCATTGATGGCTTTTACAGCTG
CTGTCACCAGCCCACTAACCACTAGCCAAACCCTCCTCTTCAACATATTGGGGGGGTGGGTGGCTGCCCAGCTCGCCGCCCCC
GGTGCCGCTACCGCCTTTGTGGGCGCTGGCTTAGCTGGCGCCGCCATCGGCAGTGTTGGACTGGGGAAGGTCCTCGTGGACAT
TCTTGCAGGGTATGGCGCGGGCGTGGCGGGAGCTCTTGTAGCATTCAAGATCATGAGCGGTGAGGTCCCCTCCACGGAGGACC
TGGTCAATCTACTGCCCGCCATCCTCTCGCCTGGAGCCCTTGTAGTCGGTGTGGTCTGCGCAGCAATACTGCGCCGGCACGTT
GGCCCGGGCGAGGGGGCAGTGCAATGGATGAACCGGCTGATAGCCTTCGCCTCCCGGGGGAACCATGTTTCCCCCACGCACTA
CGTGCCGGAGAGCGATGCAGCTGCCCGCGTCACTGCCATACTCAGCAGCCTCACTGTAACCCAGCTCCTGAGGCGACTGCACC
AGTGGATAAGCTCGGAGTGTACCACTCCATGCTCCGGTTCCTGGCTAAGGGACATCTGGGACTGGATATGCGAGGTGCTGAGC
GACTTTAAGACCTGGCTAAAAGCCAAGCTCATGCCACAACTGCCTGGGATTCCCTTTGTGTCCTGCCAGCGCGGGTATAGGGG
GGTCTGGCGAGGGGACGGCATCATGCACACTCGCTGCCACTGTGGAGCTGAGATCACTGGACATGTCAAAAACGGGACGATGA
GGATCGTCGGTCCTAAGACCTGCAGGAACATGTGGAGTGGGACCTTCCCCATTAACGCCTACACCACGGGCCCCTGTACCCCC
CTTCCTGCGCCGAACTATACGTTCGCGCTGTGGAGGGTGTCTGCAGAGGAATACGTGGAGATAAGGCAGGTGGGGGACTTCCA
CTACGTGACGGGTATGACTACTGACAATCTTAAATGCCCGTGCCAGGTCCCATCGCCCGAATTTTTCACAGAATTGGACGGGG
TGCGCCTACATAGGTTTGCGCCCCCTTGCAAGCCCTTGCTGCGGGAGGAGGTATCATTCAGAGTAGGACTCCACGAGTACCCG
GTGGGGTCGCAATTACCTTGCGAGCCCGAACCGGACGTGGCCGTGTTGACGTCCATGCTCACTGATCCCTCCCATATAACAGC
AGAGGCGGCCGGGAGAAGGTTGGCGAGGGGATCACCCCCTTCTGTGGCCAGCTCCTCGGCTAGCCAGCTGTCCGCTCCATCTC
TCAAGGCAACTTGCACCGCCAACCATGACTCCCCTGACGCCGAGCTCATAGAGGCTAACCTCCTGTGGAGGCAGGAGATGGGC
GGCAACATCACCAGGGTTGAGTCAGAGAACAAAGTGGTGATTCTGGACTCCTTCGATCCGCTTGTGGCGGAGGAGGATGAGCG
GGAGATCTCCGTACCCGCAGAAATCCTGCGGAAGTCTCGGAGATTCGCCCCGGCCCTGCCCATTTGGGCGCGGCCGGACTACA
ACCCCCCGCTGCTGGAGACGTGGAAAAAGCCTGACTACGAACCACCTGTGGTCCATGGCTGCCCGCTTCCACCTCCACAGTCC
CCTCCTGTGCCTCCGCCTCGGAAGAAGCGGACGGTGGTCCTCACCGAATCAACCGTATCTACTGCCTTGGCCGAGCTTGCCAC
CAAAAGTTTTGGCAGCTCCTCAACTTCCGGTATTACGGGCGACAATACGACAACATCCTCTGAGCCCGCCCCTTCTGGCTGCC
CCCCAGACTCCGACGCTGAGTCCTATTCTTCCATGCCCCCCCTGGAGGGGGAGCCTGGGGATCCGGATCTCAGCGACGGGTCA
TGGTCGACGGTCAGTAGTGGGGCCGACACGGAGGATGTCGTGTGCTGCTCAATGTCTTATTCCTGGACAGGCGCACTCGTCAC
CCCGTGCGCCGCGGAAGAACAAAAACTGCCCATCAACGCACTGAGCAACTCGTTGCTACGTCACCACAATCTGGTGTATTCCA
CCACCTCACGCAGTGCTTGCCAAAGGCAGAAGAAAGTCACATTTGACAGACTGCAAGTTCTGGACAGCCATTACCAGGACGTG
CTCAAGGAGGTTAAGGCAGCGGCGTCAAAAGTGAAGGCTAACTTGCTATCCGTAGAGGAAGCTTGCAGCCTGACGCCCCCACA
CTCAGCCAAATCCAAGTTTGGCTATGGGGCAAAAGACGTCCGTTGCCATGCCAGAAAGGCCGTAAACCACATCAACTCCGTGT
GGAAAGACCTTCTGGAAGACAGTGTAACACCAATAGACACTACCATCATGGCTAAGAACGAGGTTTTCTGCGTTCAGCCTGAG
AAGGGGGGTCGTAAGCCAGCTCGTCTCATCGTGTTCCCCGACCTGGGTGTGCGCGTGTGCGAGAAGATGGCCCTGTACGACGT
GGTCAGCAAGCTCCCCCTGGCCGTGATGGGAAGCTCCTACGGATTCCAATACTCACCAGGACAGCGGGTTGAATTCCTCGTGC
AAGCGTGGAAGTCCAAGAAGACCCCAATGGGGTTCTCGTATGATACCCGCTGCTTTGACTCCACAGTCACTGAGAGCGATATC
CGTACGGAGGAGGCAATCTACCAATGTTGTGACCTGGACCCCCAAGCCCGCGTGGCCATCAAGTCCCTCACTGAGAGGCTTTA
TGTTGGGGGCCCTCTTACCAATTCAAGGGGGGAGAACTGCGGCTATCGCAGGTGCCGCGCGAGCGGCGTACTGACAACTAGCT
GTGGTAACACCCTCACTTGCTACATCAAGGCCCGAGCAGCCTGTCGAGCCGCAGGGCTCCAGGACTGCACCATGCTCGTGTGT
GGCGACGACTTAGTCGTTATCTGTGAAAGTGCGGGGGTCCAGGAGGACGCGGCGAGCCTGAGAGCCTTCACGGAGGCTATGAC
CAGGTACTCCGCCCCCCCCGGGGACCCCCCACAACCAGAATACGACTTGGAGCTCATAACATCATGCTCCTCCAACGTGTCAG
TCGCCCACGACGGCGCTGGAAAGAGGGTCTACTACCTTACCCGTGACCCTACAACCCCCCTCGCGAGAGCCGCGTGGGAGACA
GCAAGACACACTCCAGTCAATTCCTGGCTAGGCAACATAATCATGTTTGCCCCCACACTGTGGGCGAGGATGATACTGATGAC
CCATTTCTTTAGCGTCCTCATAGCCAGGGATCAGCTTGAACAGGCCCTTGATTGCGAGATCTACGGAGCCTGCTACTCCATAG
AACCACTGGATCTACCTCCAATCATTCAAAGACTCCATGGCCTTAGCGCATTTTCACTCCACAGTTACTCTCCAGGTGAAATC
AATAGGGTGGCCGCATGCCTCAGAAAACTTGGGGTCCCGCCCTTGCGAGCTTGGAGACACCGGGCCCGGAGCGTCCGCGCTAG
GCTTCTGTCCAGAGGAGGCAGGGCTGCCATATGTGGCAAGTACCTCTTCAACTGGGCAGTAAGAACAAAGCTCAAACTCACTC
CAATAGCGGCCGCTGGCCAGCTGGACTTGTCCGGCTGGTTCACGGCTGGCTACAGCGGGGGAGACATTTATCACAGCGTGTCT
CATGCCCGGCCCCGCTGGTTCTGGTTTTGCCTACTCCTGCTTGCTGCAGGGGTAGGCATCTACCTCCTCCCCAACCGATGAAG
GTTGGGGTAAACACTCCGGCCTCTTAGGCCAGGTGGCTCCATCTTAGCCCTAGTCACGGCTAGCTGTGAAAGGTCCGTGAGCC
GCATGACTGCAGAGAGTGCTGATACTGGCCTCTCTGCAGATCATGT
